# Supplementary material for: Photophysical image analysis for sCMOS cameras: Noise modelling and estimation of background parameters in fluorescence-microscopy images
Source: PLoS One. 2025 Nov 4;20(11):e0335310. doi: 10.1371/journal.pone.0335310 (PMC12585066; doi:10.1371/journal.pone.0335310)
Supplement: S1 File — (PDF) [file pone.0335310.s001.pdf]

Supplementary Information
Photophysical image analysis for sCMOS cameras: Noise modelling and estimation of background parameters in fluorescence-microscopy images

Dibyajyoti Mohanta[1,2], Radhika Nambannor Kunnath☯[3], Erik Clarkson☯[1], Albertas Dvirnas[1,3], Fredrik Westerlund[3], Tobias Ambjörnsson[1]

1 Centre for Environmental and Climate Science, Lund University, Sweden
2 Department of Chemistry, The State University of New York at Buffalo, NY, USA
3 Department of Life Sciences, Chalmers University of Technology, Gothenburg, Sweden

☯These authors contributed equally to this work.

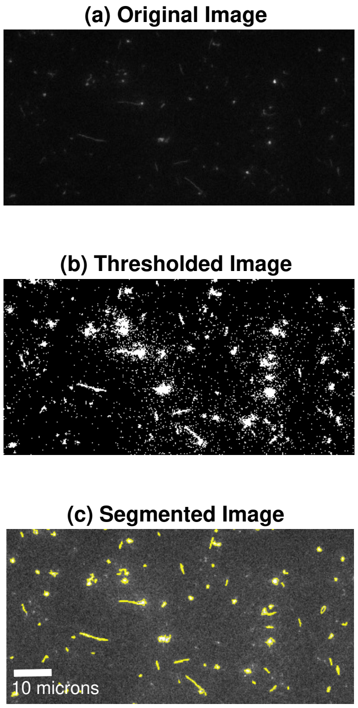

Fig S1. sCMOS pipeline performance on fluorescent DNAs on glass with 100 ms exposure time. (a) a 100 ms exposure time image of fluorescently stained DNA (balanced gain settings) (b) Binarized image processed by our unsupervised thresholding algorithm with a p-value threshold of $p_{\text{binarize}} = 0.01$. (c) Output of our segmentation approach with the yellow pixels forms the boundary of the "objects" identified by our unsupervised segmentation method. This figure is a cropped version of Fig. 3 (a) in main text (25 % at the top and 25 % at the bottom were removed for visual clarity).

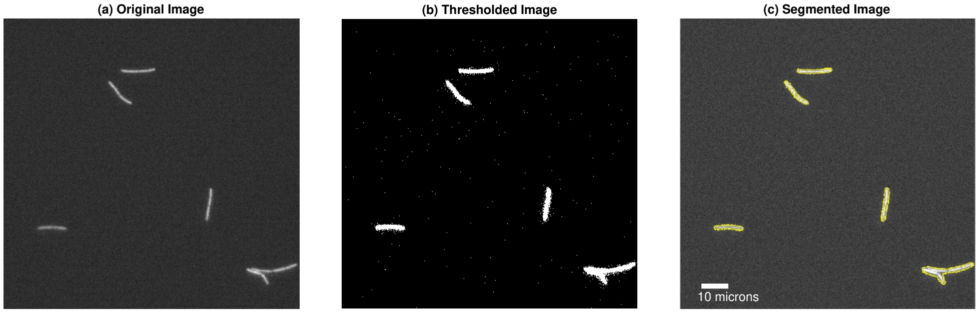

Fig S2. sCMOS pipeline performance on low exposure time (10 ms) bacteria cell images. (a) a 10 ms exposure time image of bacteria cells over expressing GFP (balanced gain settings) (b) Binarized image processed by our unsupervised thresholding algorithm with a p-value threshold of $p_{\text{binarize}} = 0.01$. (c) Output of our segmentation approach with the yellow pixels forms the boundary of the "objects" identified by our unsupervised segmentation method. Except for the exposure time, all settings were identical to the ones in Fig. 6 in the main text.

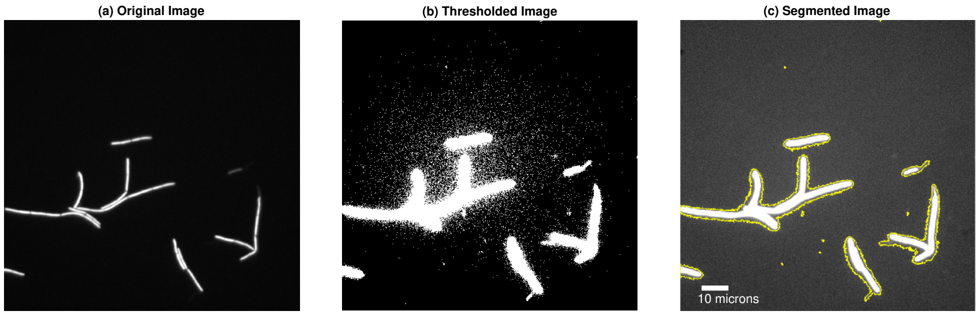

Fig S3. sCMOS pipeline performance on high exposure time (400 ms) bacteria cell images. (a) a 400 ms exposure time image of bacteria cells over expressing GFP (balanced gain settings) (b) Binarized image processed by our unsupervised thresholding algorithm with a p-value threshold of $p_{\text{binarize}} = 0.01$. (c) Output of our segmentation approach with the yellow pixels forms the boundary of the "objects" identified by our unsupervised segmentation method. Except for the exposure time, all settings were identical to the ones in Fig. 6 in the main text.
