# Supplementary material for: Photophysical image analysis for sCMOS cameras: Noise modelling and estimation of background parameters in fluorescence-microscopy images
Source: PLoS One. 2025 Nov 4;20(11):e0335310. doi: 10.1371/journal.pone.0335310 (PMC12585066; doi:10.1371/journal.pone.0335310)
Supplement: Attachment — Submitted filename: Response_to_Reviewers.pdf [file pone.0335310.s002.pdf]

Reply to Referees

1 Reviewer 1

Comment: This manuscript presents a well-structured and clearly written framework for unsupervised probabilistic image thresholding in sCMOS cameras. The derivation of the sCMOS-specific probability mass function (PMF) is rigorous, and the camera calibration procedures are carefully designed. The experimental results, including comparisons with EMCCD under matched conditions, are convincing and well illustrated.

Reply: We thank reviewer for the critical evaluation of the manuscript and generous comments regarding publication of this manuscript in PLOS One.

Comment:One minor concern is the potential impact of photobleaching in the dual-camera experiment (Figure 7), as the sCMOS images were acquired before EMCCD. While the authors mention steps to mitigate bleaching, it would be helpful to clarify whether the acquisition order was counterbalanced or to briefly discuss the lower λ_{bg} observed for EMCCD at higher exposure times.

Reply: In the revised manuscript (subsection "Experiments"), we clarified the order at which the images were acquired (if an image was captured at one exposure setting first with sCMOS and then EMCCD, the image for the next exposure setting was captured first with EMCCD and then sCMOS). We also elaborated on potential causes for why the estimates of $\lambda_{bg}/(\text{pixel area})$ are slightly different for the two camera types (please see the discussion at the end of the Results section).

2 Reviewer 2

Comment: I appreciated the updated manuscript that included the full reference list and placement of the Figures within the main text. It was very helpful to properly review the manuscript. The authors have a history of publishing manuscripts to improve the quality of fluorescence microscopy images for analysis. This current study on photophysical image analysis (PIA) for sCMOS cameras is a logical extension of their previous work that introduced PIA with unsupervised probabilistic image thresholding for images acquired by EMCCD

cameras. The manuscript is well written, and the authors present the information in a clear manner. This work is suitable for publication in the journal of PLoS One with revisions.

Reply: We thank the reviewer for reading our manuscript and for recommending publication of this manuscript in PLOS One after revisions.

Comment: My major concern is that it would be highly beneficial to include 1-2 more figures (either in the main text or supplemental) to document the application of their technique to a variety of images. It is difficult to appreciate the image processing pipeline and segmentation from the images in Figure 6. Consider adding Figures like Figures 3 and S10 from their 2024 PLoS One publication, or like Figures from references 2 (Nature Methods. 2017) or 3 (Nature Comm 2020) cited in this manuscript.

Reply: We recorded images of stained bacterial cells fixed on a glass side, and applied our analysis pipeline to these. In the revised manuscript, one of these images (100 ms exposure time) replaced the image of DNA on glass (which is now placed in the Supplementary). Further images (low and high exposure times images) are shown in the Supplementary. We thank the reviewer for this suggestion, which shows the versatility of our method.

Comment: The 2nd and 3rd authors are designated as contributing equally to this work. Was the first author also an equal contributor, or just the 2nd and 3rd author?

Reply: The first author is a "sole" first author. it is only the 2nd and 3rd authors which contributed equally. Thus, authors order and equal contributions were correctly noted in the previously submitted version.
Comment: Figure 2. The location of (a) and (b) labels should be moved to the upper left-hand corner inside or outside the graph or by the graph title (like Figure 3).
Reply:
We have corrected the labels.

Comment: Figure 3. The locations of tiles 7,13 and 8,8 in (a) are unclear. It would be helpful to mark these tiles with different colored outlines to make it easier for the reader to compare the histogram in (b) to tile 7,13 and the histogram in (c) to tile 8,8.
Reply: We have marked the tiles 7,13 and 8,8 with yellow and green border respectively. We thank the reviewer for this suggestion, which improved clarity.

Comment: Figure 4. The location of (a), (b), and (c) labels should be moved to the upper left-hand corner inside or outside the graph or by the graph title (like Figure 3).
Reply: We have corrected Figure 4. labels.

Comment: There are at least 2 reference that are incorrect (see below). Please carefully review all references for accuracy.

Reference 3 (line 470) is cited as "Nature Communications. 2020;11:238" and should be "Nature Communications. 2020;11:94"

Reference 10 (lines 489-490) is cited as "PLoS ONE. 2024;19(4):e0298321" and should be "PLoS ONE. 2024;19(4): e0300122"

Reply: Thanks for the particular comment. We have rectified the references.
